# Effect of Alumina Airborne-Particle Abrasion Followed by Plasma Treatment on Bond Strength of Dental PEEK to MMA-Based Luting Systems

**DOI:** 10.3390/bioengineering13050507

**Published:** 2026-04-28

**Authors:** Taro Mukaibo, Takafumi Watanabe, Ayako Miura, Kanna Saimoto, Misaki Matsuo, Hiromichi Ogusu, Chihiro Masaki, Hiroshi Ikeda

**Affiliations:** 1 Division of Oral Reconstruction and Rehabilitation, Kyushu Dental University, Kitakyushu 803-8580, Japan; r07mukaibou@fa.kyu-dent.ac.jp (T.M.); r21miura@fa.kyu-dent.ac.jp (A.M.); masaki@kyu-dent.ac.jp (C.M.); 2 Division of Occlusion and Maxillofacial Reconstruction, Kyushu Dental University, Kitakyushu 803-8580, Japan; r17ogusu@fa.kyu-dent.ac.jp; 3 Division of Biomaterials, Kyushu Dental University, Kitakyushu 803-8580, Japan; r25saimoto@fa.kyu-dent.ac.jp (K.S.); r23matsuo2@fa.kyu-dent.ac.jp (M.M.)

**Keywords:** poly (ether ether ketone), surface modification, plasma, sandblast, bond strength, resin cement

## Abstract

Poly (ether ether ketone) (PEEK) has attracted increasing attention for dental applications because of its favorable mechanical properties, physicochemical stability, and biocompatibility. However, its inherently poor bonding characteristics remain a major limitation in clinical practice. This study investigated the effect of sequential alumina airborne-particle abrasion (sandblasting) followed by plasma treatment on the bonding performance of methyl methacrylate (MMA)-based luting systems to dental CAD-CAM PEEK. PEEK specimens were prepared as plates and divided into four surface-treatment groups: untreated, airborne-particle abraded, plasma-treated, and airborne-particle abraded followed by plasma treatment. Surface characteristics were evaluated using SEM–EDX analysis and surface roughness measurements, and surface wettability was assessed by contact angle measurements using primers from two MMA-based luting systems (Beautylink [BL] and Super-Bond [SB]). Shear bond strength (SBS) between treated PEEK and each luting system was determined after 24 h of water storage (initial) and after 20,000 thermocycles (aged). Airborne-particle abrasion significantly increased surface roughness, whereas plasma treatment enhanced surface wettability without altering roughness. The combined treatment resulted in the highest surface roughness and the lowest contact angles and demonstrated superior or comparable SBS compared with the single treatments. After aging, the combined treatment significantly improved bonding durability. These findings indicate that airborne-particle abrasion followed by plasma treatment enhances the bonding performance and durability of MMA-based luting systems to PEEK.

## 1. Introduction

Poly (ether ether ketone) (PEEK) is a high-performance thermoplastic polymer that has attracted increasing attention for dental applications because of its favorable mechanical properties, physicochemical stability, and biocompatibility [1]. PEEK exhibits high toughness and impact resistance, contributing to resistance against fracture under force loading [2,3]. In addition, its relatively low elastic modulus compared with ceramics and metals may provide a stress-mitigating “cushioning” effect under functional loading [4]. PEEK also demonstrates excellent chemical stability and high resistance to water absorption, dissolution, and discoloration or staining, supporting its use in the oral environment [5,6]. From a biological perspective, PEEK exhibits minimal elution of unreacted monomers or additives, resulting in excellent biocompatibility and low irritation potential for oral mucosa and gingival tissues [7,8]. Moreover, the hydrophobic surface and low surface free energy of PEEK have been reported to reduce bacterial adhesion and plaque accumulation [9]. Owing to these characteristics, dental-grade PEEK has been applied to a wide range of prosthodontic and implant-related indications, including crowns, bridges, implant components, endodontic posts, removable prostheses, and orthodontic devices [10,11].

Despite these advantages, reliable adhesion to PEEK remains a major clinical challenge [12]. PEEK is chemically inert and lacks reactive functional groups, resulting in limited chemical interaction with conventional dental adhesive systems [13]. Consequently, insufficient bonding to luting agents may lead to debonding of PEEK restorations and compromise long-term clinical outcomes. To address this limitation, extensive research has investigated surface pretreatments and adhesive strategies for improving bonding to PEEK [14]. Among dental adhesive systems, methyl methacrylate (MMA)-containing primers and luting agents have been reported to provide higher bond strength and improved durability [15]. Several studies have demonstrated that MMA-containing primers, such as Signum PEEK Bond^®^ and Visio.link^®^, enhance the bonding of PEEK to resin-based veneering materials [16,17]. In addition, MMA-based luting systems (resin cements), such as Super-Bond^®^ and Block HC Cem^®^, when used in combination with MMA-containing primers, have shown more favorable bonding performance to PEEK than composite-based resin cements [18,19]. These findings indicate that MMA-based luting systems are promising options for achieving reliable adhesion to PEEK.

Surface pretreatment of PEEK is a critical determinant of bonding effectiveness. Numerous physical and chemical surface modification methods have been evaluated, including airborne-particle abrasion (sandblasting) [20,21], plasma treatment [22,23], ultraviolet irradiation [24,25], laser treatment [26,27], chemical etching (e.g., sulfuric acid) [28,29], and various coating approaches [30,31]. Among these methods, both high-concentration sulfuric acid etching and alumina airborne-particle abrasion have been reported to effectively increase surface roughness and promote micromechanical interlocking, thereby enhancing bond strength. However, the clinical applicability of concentrated sulfuric acid is limited due to safety and handling concerns, making airborne-particle abrasion a more practical pretreatment for routine clinical use.

Based on current evidence, a commonly recommended approach for PEEK bonding is airborne-particle abrasion followed by the application of an MMA-based luting system [32]. However, airborne-particle abrasion alone primarily provides micromechanical retention and does not sufficiently increase surface energy or introduce reactive functional groups on the PEEK surface. This limitation may restrict effective interaction with MMA-based luting systems. Consequently, debonding of PEEK restorations, such as crowns and bridges, remains a clinical concern, indicating that further improvement in bonding effectiveness and durability is required for long-term prosthetic success.

To further modify the airborne-particle-abraded PEEK surface, plasma treatment has attracted attention as an additional surface modification method because it can increase surface energy and introduce polar functional groups without the use of hazardous chemicals [33]. Therefore, plasma treatment is expected to complement the limitations of airborne-particle abrasion by enhancing surface wettability and chemical affinity without altering the microretentive morphology.

The purpose of this study was to evaluate the effect of combined alumina airborne-particle abrasion and plasma treatment on the bond strength between dental PEEK and an MMA-based luting system. The null hypotheses were that additional plasma treatment after airborne-particle abrasion would not affect the surface roughness and wettability of PEEK, and would not improve the bond strength between PEEK and MMA-based luting systems.

## 2. Materials and Methods

### 2.1. Surface Treatment of PEEK

**Figure 1:**
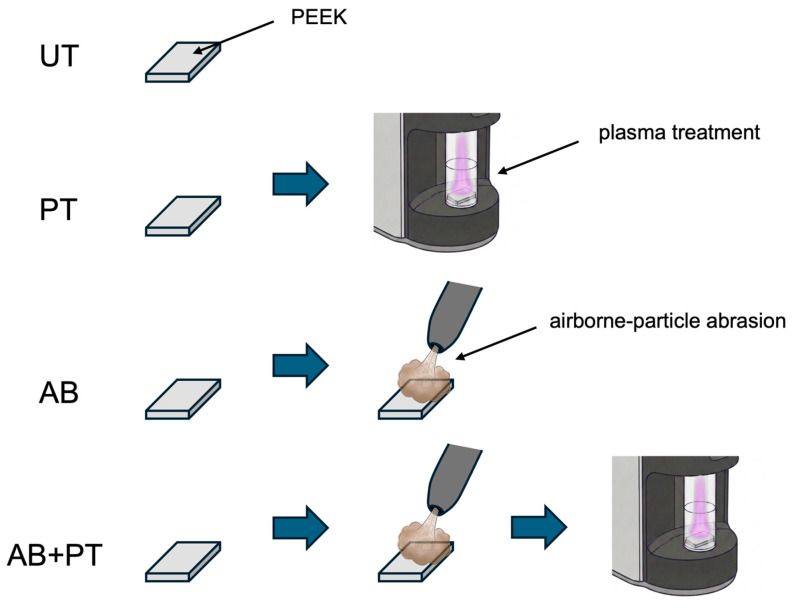
Schematic overview of the experimental procedure.

A schematic overview of the experimental procedure is presented in Figure 1. Two commercial MMA-based luting systems were employed in this study (Table 1). Beautylink (BL) is a dual-cure, composite-based resin cement used in combination with an MMA-containing primer. Super-Bond (SB) is a self-cure, MMA-based resin cement also used with an MMA-containing primer.

Commercial CAD-CAM PEEK blocks (Table 2) were sectioned into plates with a thickness of 2 mm using a diamond wheel saw. The plates were sequentially polished with #600 emery papers. After polishing, a total of 244 specimens were ultrasonically cleaned in distilled water for 5 min and air-dried. The prepared specimens were then randomly assigned to four surface-treatment groups (*n* = 61 per group) as summarized in Table 3:

• Untreated group (UT): No additional surface treatment was performed.
• Plasma-treated group (PT): Plasma treatment was applied to the PEEK surface using a dental plasma device (ACTILINK Reborn, AOS Technology, Plasmapp Co., Ltd., Seoul, Republic of Korea) for 60 s in an air atmosphere at 5–10 Torr.
• Airborne particle abraded group (AB): Airborne-particle abrasion was performed on the PEEK surface using 50 μm alumina particles (purity 95%, Akiyama Sangyo Co., Ltd., Osaka, Japan) with an airborne-particle abrader (Jet Blast II, J. Morita Corp., Suita, Japan) at an air pressure of 0.2 MPa for 10 s and a nozzle-to-surface distance of 10 mm. Residual alumina particles were removed by air blowing.
• Airborne particle abraded + plasma-treated group (AB + PT): Airborne-particle abrasion was first performed as described above, followed by plasma treatment under the same conditions.

These specimens were subjected to the following evaluations.

### 2.2. SEM-EDX Observation

Surface morphology and elemental analysis of the specimens were examined using scanning electron microscopy with energy dispersive X-ray spectroscopy (SEM–EDX; ERA-600, ELIONIX INC., Tokyo, Japan) at an accelerating voltage of 15 kV (*n* = 1 per group). Prior to observation, the specimen surfaces were sputter-coated with gold.

### 2.3. Surface Roughness Measurement

Surface roughness (Ra) was measured using a stylus profilometer (HANDYSURF+, Tokyo Seimitsu Co., Ltd., Tokyo, Japan). Measurements were performed at the center of each specimen over a 10 mm tracing length. Ten independent specimens were measured once for each group (*n* = 10).

### 2.4. Contact Angle Measurement

The wettability of each treated PEEK surface was evaluated by measuring the contact angle of the primers from the MMA-containing luting systems using the static sessile drop method with a contact angle meter (DMe-211, Kyowa Interface Science Co., Ltd., Saitama, Japan) at 25 °C. Droplet images were captured digitally and analyzed using the dedicated software. Ten independent specimens were measured once for each group (*n* = 10).

### 2.5. Shear Bond Strength (SBS) Test

Bonding performance between treated PEEK surfaces and MMA-based luting systems was evaluated using a shear bond strength (SBS) test according to our previous protocol [34]. A Teflon tube (inner diameter: 5 mm) was fixed onto the PEEK surface using double-sided tape to standardize the bonding area. After primer application, the luting agent was filled into the tube. For the BL luting system (dual-cure resin cement), light polymerization was performed using a light-curing unit (α LIGHT II N, J. Morita, Osaka, Japan), followed by storage at 25 °C for 1 h. For the SB luting system (self-cure resin cement), specimens were stored at 25 °C for 1 h without light irradiation. After polymerization, the Teflon tube was removed, and specimens were immersed in distilled water at 37 °C for 24 h. These specimens were defined as the initial group. For bonding durability evaluation, additional specimens were subjected to 20,000 thermocycles between 5 °C and 55 °C with a dwell time of 60 s in each bath using a thermocycling device (K178, Tokyo Giken, Tokyo, Japan). These specimens were defined as the aged group. SBS was measured using a universal testing machine (AGS-H, Shimadzu, Kyoto, Japan) at a crosshead speed of 1.0 mm/min. The sample size (*n* = 10 per group) was determined based on previous similar studies on PEEK bonding [18]. Failure modes were observed under optical microscopy at ×30 magnification and classified as: (1) adhesive failure at the PEEK–cement interface; (2) cohesive failure within the luting agent; (3) mixed failure of adhesive and cohesive.

### 2.6. Statistical Analysis

All statistical analyses were performed using R version 4.5.2 (R Foundation for Statistical Computing, Vienna, Austria). Surface roughness and contact angle data were analyzed separately for each luting system (BL and SB) using one-way analysis of variance (ANOVA), followed by Tukey’s post hoc test for multiple comparisons among surface-treatment groups. SBS data were analyzed separately for each luting system using two-way ANOVA to evaluate the effects of surface treatment (four levels: untreated, airborne-particle abrasion, plasma-treated, and airborne-particle abrasion followed by plasma treatment) and aging condition (two levels: initial and aged), as well as their interaction. When significant interactions were detected, simple main effects were further examined, and multiple comparisons were conducted using Tukey’s test. Statistical significance was set at *p* < 0.05.

## 3. Results

### 3.1. Surface Modification of PEEK

Surface modifications of PEEK induced by plasma treatment, airborne-particle abrasion, and their combination were evaluated by SEM–EDX analysis, surface roughness measurement, and contact angle measurement using primers of MMA-based luting systems.

**Figure 2:**
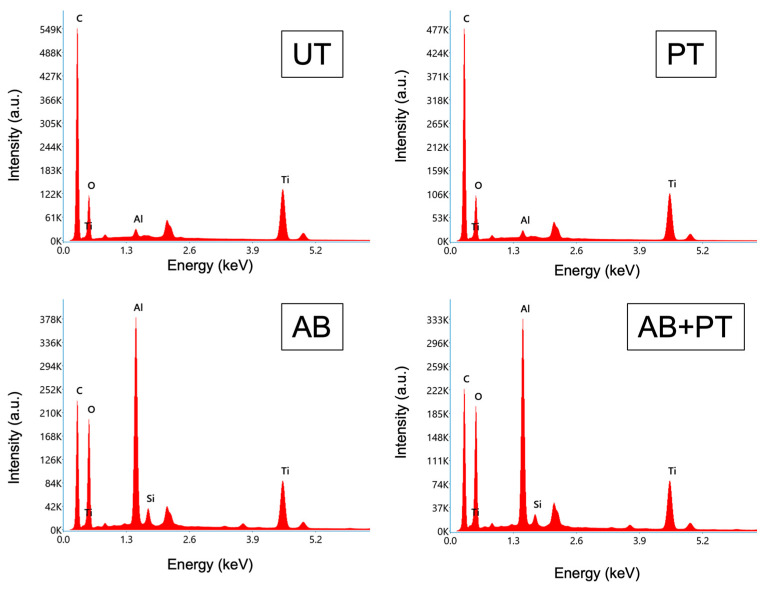
EDX spectra obtained from the treated PEEK surfaces: untreated (UT), plasma-treated (PT), airborne-particle abrasion (AB), and airborne-particle abrasion followed by plasma treatment (AB + PT). The spectra were acquired at ×1000 magnification using SEM–EDX analysis.

Figure 2 shows the EDX spectra of the treated PEEK surfaces. The spectrum of the UT specimen exhibited peaks corresponding to carbon and oxygen derived from the PEEK matrix, as well as titanium and oxygen originating from the TiO_2_ filler incorporated in the material. PT did not produce noticeable changes in the spectral profile. In contrast, AB altered the spectrum by introducing additional peaks corresponding to aluminum and silicon, which were attributed to alumina particles and trace silica impurities originating from the airborne-particle abrasion process. The spectrum of the AB + PT specimen was similar to that of the AB specimen.

**Figure 3:**
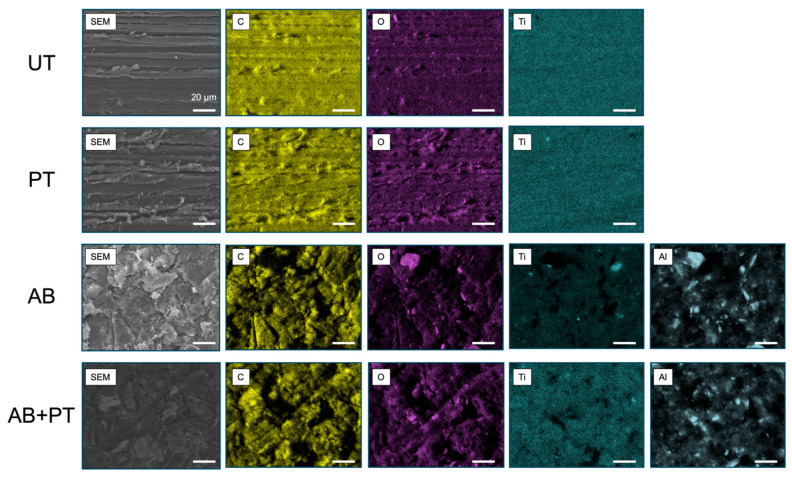
SEM images and corresponding elemental maps (C, O, Ti, and Al) of treated PEEK surfaces: untreated (UT), plasma-treated (PT), airborne-particle abrasion (AB), and airborne-particle abrasion followed by plasma treatment (AB + PT). Images were acquired at ×1000 magnification using SEM–EDX. Scale bar: 20 μm.

Figure 3 presents SEM images and the corresponding elemental maps of the treated PEEK surfaces. The UT specimen exhibited surface scratches caused by the polishing process. Plasma treatment did not produce noticeable changes in surface morphology compared with the untreated surface. In contrast, airborne-particle abrasion generated a roughened surface characterized by randomly distributed concave–convex microstructures. A similar roughened morphology was observed in the AB + PT specimens. Elemental mapping of the UT specimen showed that carbon, oxygen, and titanium were homogeneously distributed across the surface, confirming the presence of TiO_2_ fillers dispersed within the PEEK matrix. Plasma treatment did not significantly alter the elemental distribution. In contrast, the AB specimen exhibited aluminum signals distributed across the surface, indicating the presence of residual alumina particles originating from the abrasion process. This tendency was also observed in the AB + PT specimens.

**Figure 4:**
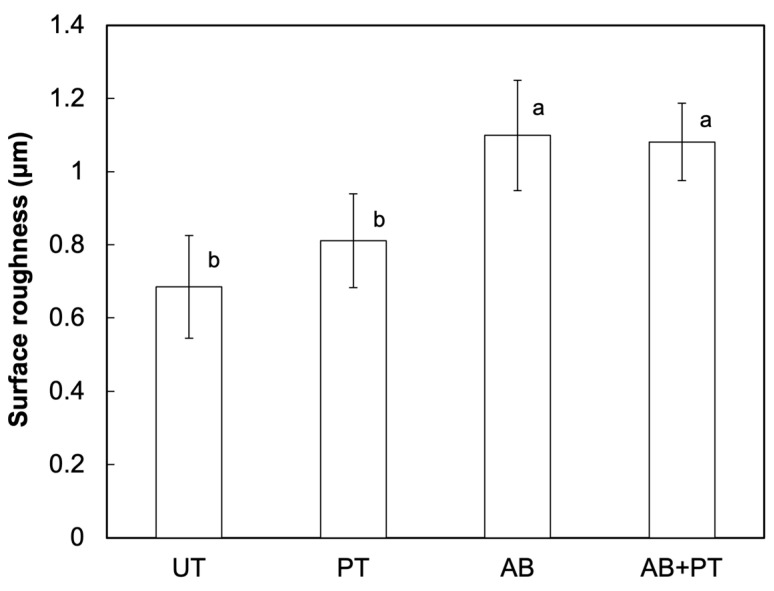
Surface roughness (Ra) of treated PEEK specimens: untreated (UT), plasma-treated (PT), airborne-particle abrasion (AB), and airborne-particle abrasion followed by plasma treatment (AB + PT). Different letters indicate statistically significant differences among groups.

Surface roughness was quantitatively evaluated by measuring the average roughness (Ra) (Figure 4). PT did not significantly change the surface roughness compared with UT. In contrast, AB significantly increased the surface roughness. The combined treatment (AB + PT) also resulted in a significant increase in roughness compared with the UT group, while no significant difference was observed compared with the AB group. These results indicate that plasma treatment did not affect the surface roughness of the abraded PEEK surface.

**Figure 5:**
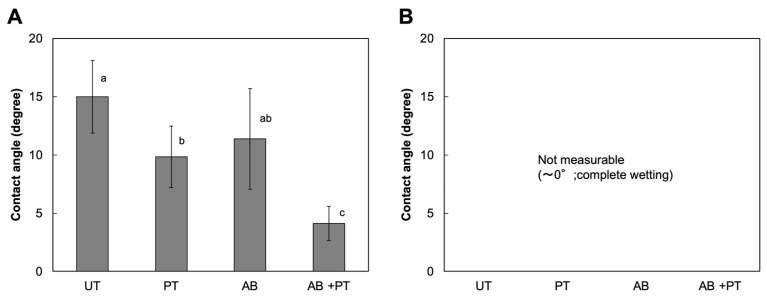
Contact angles of primers from MMA-based luting systems on treated PEEK surfaces: (**A**) BL primer and (**B**) SB primer. The contact angle of the SB primer was not measurable due to its extremely low contact angle (near-complete spreading). UT: untreated; PT: plasma-treated; AB: airborne-particle abrasion; AB + PT: airborne-particle abrasion followed by plasma treatment. Different letters indicate statistically significant differences among groups.

Changes in surface properties were further evaluated by measuring the wettability of treated PEEK surfaces to the primers (Figure 5). For the BL primer, plasma treatment significantly reduced the contact angle compared with the untreated surface. AB also reduced the contact angle. Notably, the combined treatment resulted in a significantly lower contact angle than either PT or AB. These findings demonstrate that the combination of airborne-particle abrasion and plasma treatment resulted in greater improvement in surface wettability than either treatment alone.

For the SB primer, the contact angle could not be measured in any group because the values were too low to be accurately determined.

### 3.2. Bonding Performance of Treated PEEK to MMA-Based Luting Systems

Bonding performance between treated PEEK surfaces and MMA-based luting systems (BL and SB) was evaluated using the shear bond strength (SBS) test under initial and aged conditions.

**Figure 6:**
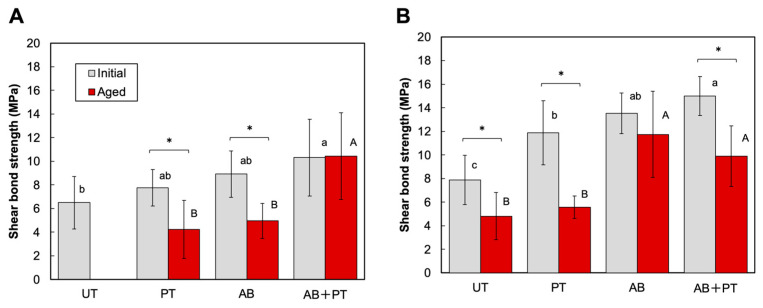
Shear bond strength (SBS) between treated PEEK surfaces and MMA-based luting systems: (**A**) BL and (**B**) SB. UT: untreated; PT: plasma-treated; AB: airborne-particle abrasion; AB + PT: airborne-particle abrasion followed by plasma treatment. Different letters indicate statistically significant differences among groups. An asterisk (*) indicates a statistically significant difference between the initial and aged groups within the same surface treatment (*p* < 0.05, Tukey’s test).

For the BL system, two-way ANOVA revealed significant effects of surface treatment (*p* < 0.001, η^2^p = 0.400), aging condition (*p* = 0.003, η^2^p = 0.128), and a significant interaction between surface treatment and aging condition (*p* = 0.022, η^2^p = 0.114). Because a significant interaction was observed, simple main effects were further analyzed. As shown in Figure 6A, no significant differences in SBS were observed among the treatment groups under the initial condition. However, after aging, significant differences were detected among the treatments. The AB + PT group exhibited significantly higher SBS than the PT and AB groups, whereas the UT group showed complete debonding after thermocycling. These results indicate that the combined treatment markedly improved bonding durability in the BL system.

**Figure 7:**
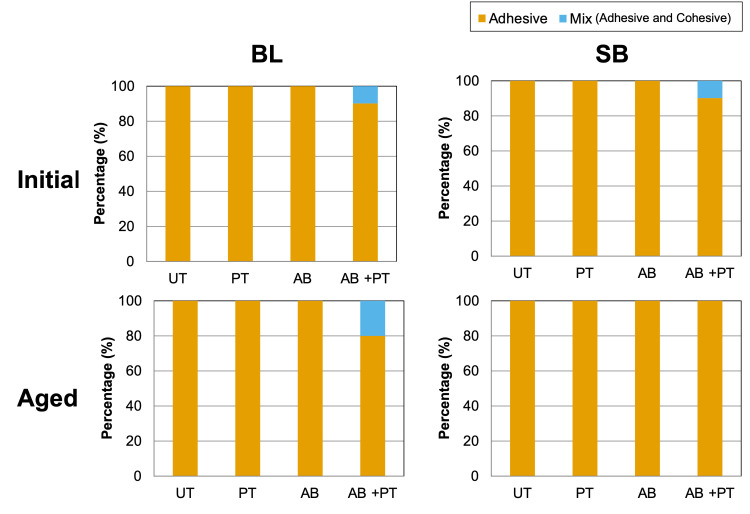
Failure modes observed after the shear bond strength test: BL and SB. UT: untreated; PT: plasma-treated; AB: airborne-particle abrasion; AB + PT: airborne-particle abrasion followed by plasma treatment.

For the SB system, two-way ANOVA also demonstrated significant effects of surface treatment (*p* < 0.001, η^2^p = 0.595), aging condition (*p* < 0.001, η^2^p = 0.466), and their interaction (*p* = 0.012, η^2^p = 0.139). Because the interaction was significant, simple main effects were examined. As shown in Figure 6B, under the initial condition, the AB + PT group exhibited significantly higher SBS than the UT group. After aging, the AB and AB + PT groups showed significantly higher SBS than the UT group, whereas the PT group did not maintain bonding performance comparable to the AB group. These findings suggest that AB plays a dominant role in bonding durability in the SB system. Failure mode analysis (Figure 7) revealed predominantly adhesive failures in all groups. However, mixed failures were observed in a small number of specimens in the AB + PT group: one specimen in the BL system and one specimen in the SB system under the initial condition, and two specimens in the BL system under the aged condition.

## 4. Discussion

This study evaluated the effect of airborne-particle abrasion followed by plasma treatment on the bonding performance of MMA-based luting agents to PEEK using two systems, BL and SB. The results demonstrated that airborne-particle abrasion provided effective micromechanical roughening, whereas subsequent plasma treatment enhanced surface wettability. As a result, the combined treatment improved bonding performance, particularly bonding durability. Although the degree of improvement varied between the BL and SB systems, the combined treatment exhibited superior or comparable shear bond strength (SBS) relative to the single treatments. These findings suggest that the combined effect of mechanical interlocking and chemical activation contributes to enhanced bonding durability. Accordingly, the null hypotheses were rejected in part. Additional plasma treatment after airborne-particle abrasion did not significantly alter the surface roughness of PEEK, but it improved surface wettability and enhanced bonding performance, particularly bonding durability.

Alumina airborne-particle abrasion in the present study caused impact and abrasion of the PEEK surface by alumina particles, generating micrometer-scale grooves and irregular structures. These surface features allow penetration of the MMA-based luting agent, which subsequently polymerizes within the microgrooves, forming a mechanical anchoring structure at the interface. This micromechanical interlocking is considered to be the primary reason for the increased bond strength observed after airborne-particle abrasion. Similar interlocking effects induced by alumina airborne-particle abrasion have been widely reported in bonding between luting agents and PEEK, contributing to enhanced bond strength [31,35,36]. The present results further indicate that alumina particles remained on the PEEK surface after airborne-particle abrasion, as confirmed by SEM–EDX analysis. These residual particles could not be removed by air blowing, and additional attempts to eliminate them by ultrasonication were also unsuccessful, suggesting that they were tightly adhered to the PEEK surface. Such retained particles may influence the adhesive characteristics of abraded PEEK. Although not specific to PEEK, previous studies have reported retention of alumina particles on metal alloy surfaces after airborne-particle abrasion [37], and these particles have been suggested to affect bonding characteristics [38,39]. Retained alumina particles may contribute positively by modifying the surface condition, but they could also act as an unstable intermediate layer depending on their distribution and retention. However, the extent and direction of this influence were not clarified in the present study, and further investigation is required to elucidate the effect of residual alumina on PEEK adhesion.

Plasma treatment effectively improved the wettability of PEEK to the primers and enhanced bonding to the luting agents. This change in surface properties suggests that plasma treatment may have introduced reactive functional groups onto the PEEK surface. In the present study, plasma treatment was performed in an air atmosphere, generating oxygen-containing species at the surface. These reactive plasma species can remove surface contaminants and introduce polar functional groups, such as –C=O, –COOH, and –OH, onto the PEEK surface [33,40,41]. Such polar groups are capable of participating in chemical interactions with functional components of adhesive systems, including silane coupling agents, potentially forming Si–O–C bonds [23,42]. This chemical modification of the PEEK surface by plasma treatment may contribute to the improved bonding performance observed between PEEK and MMA-based luting agents. It should be noted, however, that contact angle measurement for the SB primer was not feasible in any group due to immediate spreading upon contact, which prevented stable droplet formation. Consequently, quantitative wettability data could not be obtained for the SB system, and interpretations regarding the role of surface wettability in SB bonding performance should, therefore, be made with caution.

Sequential airborne-particle abrasion and plasma treatment, therefore, produced complementary surface modifications on PEEK, whereby the surface was mechanically roughened by airborne-particle abrasion and chemically activated by plasma treatment. These complementary effects contributed to enhanced interfacial adhesion. Notably, the superior performance observed after aging suggests that chemical activation by plasma treatment may contribute to improved interfacial stability against hydrolytic degradation. The combined utilization of micromechanical interlocking and chemical surface modification appears to be particularly important for bonding durability. In the present study, the mixed failures observed in the AB + PT groups suggest that the interfacial bond strength locally approached the cohesive strength of the materials, evidencing improved adhesion. However, the predominance of adhesive failure overall confirms that the interface remains the “weakest link,” indicating that further optimization of PEEK bonding is still possible.

A comparable concept has been demonstrated in previous studies reporting improved bonding performance of PEEK through sequential surface treatments [23,43,44,45,46]. For example, sulfonic acid etching followed by plasma treatment improved bond strength between treated PEEK and resin materials via silane coupling agents [23]. Similarly, piranha solution etching followed by airborne-particle abrasion increased surface area and surface reactivity, leading to enhanced bonding performance [46]. Although these studies support the effectiveness of sequential mechanical and chemical modifications, to the best of our knowledge, limited information has been available regarding the durability of bonding to MMA-based luting systems after combined airborne-particle abrasion and plasma treatment, and the present study extends existing knowledge by demonstrating that this combined treatment enhances bonding durability.

The combination of airborne-particle abrasion and plasma treatment is clinically feasible because both procedures are simple and require only short processing times. These advantages suggest that the approach may be a promising candidate for integration into daily clinical practice. Airborne-particle abraders and plasma-treatment devices are commercially available and already used in dental practice, making them suitable for chairside application. Moreover, these methods present fewer safety concerns than chemical etching procedures, such as concentrated sulfuric acid or hydrofluoric acid treatment. Thus, the combined approach represents a practical strategy with a low barrier to clinical implementation. Its application may reduce debonding failures of dental PEEK restorations and contribute to their long-term clinical success. Regarding clinical optimization of this approach, it should be noted that the increased surface energy of plasma-treated polymer surfaces is known to gradually decrease over time [47]. To ensure maximum efficacy in a clinical setting, it is recommended that the plasma treatment be performed chairside, immediately before the luting procedure. This workflow ensures that the PEEK surface remains in its most active state during bonding. However, several limitations of this study should be noted. First, although the shear bond strength (SBS) test employed in this study is less prone to technical errors during specimen preparation and allows for highly reproducible evaluations, it is known to produce non-uniform stress distributions at the interface. These distributions often involve tensile or peeling components that may not fully reflect clinical loading conditions. Future studies using micro-tensile bond strength tests or more complex loading models may provide further insights. Second, the aging protocol employed in this study was limited to thermocycling, which does not fully replicate clinical conditions. In particular, cyclic mechanical loading (fatigue) was not incorporated, and its absence may affect the assessment of long-term bond performance. Third, SEM–EDX analysis in the present study was limited to qualitative assessment. Future studies incorporating mechanical fatigue testing and quantitative surface characterization would provide a more comprehensive evaluation of bonding durability under clinically relevant conditions.

## 5. Conclusions

The combined treatment of airborne-particle abrasion followed by plasma treatment significantly increased surface roughness and wettability where measurable. Consequently, this combination improved the bond strength and durability between PEEK and MMA-based luting agents. Therefore, the sequential application of airborne-particle abrasion and plasma treatment in conjunction with MMA-based luting systems represents a promising approach for enhancing the long-term clinical success of PEEK restorations.

## Figures and Tables

**Table 1:** MMA-based luting systems used for experiment.

System	Manufacturer	Material	Composition
BL	Shofu Inc., Kyoto, Japan	CAD/CAM resin adhesive	MMA, UDMA, acetone, initiator, others
		Beutilink SA	Fillers, UDMA, Bis-GMA, Initiator, others
SB	Sun Medical Co. Ltd., Moriyama, Japan	M&C Primer	MMA, acetone, γ-MPTS, MDP,
		Super-Bond EX	MMA, PMMA, 4-META, TBB-O, others

CAD/CAM: computer-aided design and computer-aided manufacturing, MMA: methyl methacrylate, UDMA: urethane dimethacrylate, Bis-GMA: bisphenol A diglycidyl-methacrylate, 4-META: 4-methacryloxyethyl trimellitic anhydride, MDP: 10-methacryloyloxydecyl dihydrogen phosphate, γ-MPTS: 3-methacryloxypropyl trimethoxy silane, PMMA: polymethyl methacrylate, TBB-O: partially oxidized tri-n-butyl borane.

**Table 2:** PEEK block for CAD-CAM used in experiment.

Brand Name	Manufacturer	Condition
Shofu Block PEEK	Shofu Inc., Kyoto, Japan	Poly (ether ether ketone), TiO_2_, others

**Table 3:** Surface treatment devices and their operation condition for PEEK.

Method	Device	Manufacturer	Condition
Plasma treatment	ACTILINK Reborn	Plasmapp Co., Ltd., Seoul, Republic of Korea	60 s in an air atmosphere at 5–10 Torr.
Airborne-particle abrasion	Jet Blast II	J. Morita Corp., Suita, Japan	0.2 MPa for 10 s with 50-μm alumina particles

## Data Availability

The original contributions presented in this study are included in the article. Further inquiries can be directed to the corresponding author.
